# Report on fluorescence lifetime imaging using multiphoton laser scanning microscopy targeting sentinel lymph node diagnostics

**DOI:** 10.1117/1.JBO.25.7.071204

**Published:** 2020-03-14

**Authors:** Jeemol James, Despoina Kantere, Jonas Enger, Jan Siarov, Ann Marie Wennberg, Marica B. Ericson

**Affiliations:** a University of Gothenburg, Biomedical Photonics Group, Department of Chemistry and Molecular Biology, Gothenburg, Sweden; b University of Gothenburg, Institute of Clinical Sciences, Department of Dermatology and Venereology, Gothenburg, Sweden; c University of Gothenburg, Department of Physics, Gothenburg, Sweden; d University of Gothenburg, Department of Pathology, Gothenburg, Sweden

**Keywords:** malignant melanoma, sentinel lymph node, multiphoton microscopy, fluorescence lifetime imaging, autofluorescence

## Abstract

**Significance:** Sentinel lymph node (SLN) biopsy is an important method for metastasis staging in, e.g., patients with malignant melanoma. Tools enabling prompt histopathological analysis are expected to facilitate diagnostics; optical technologies are explored for this purpose.

**Aim:** The objective of this exploratory study was to investigate the potential of adopting multiphoton laser scanning microscopy (MPM) together with fluorescence lifetime analysis (FLIM) for the examination of lymph node (LN) tissue *ex vivo*.

**Approach:** Five LN tissue samples (three metastasis positive and two negative) were acquired from a biobank comprising tissues from melanoma patients. Tissues were deparaffinized and subjected to MPM-FLIM using an experimental MPM set-up equipped with a time correlated single photon counting module enabling FLIM.

**Results:** The data confirm that morphological features similar to conventional histology were observed. In addition, FLIM analysis revealed elevated morphological contrast, particularly for discriminating between metastatic cells, lymphocytes, and erythrocytes.

**Conclusions:** Taken together, the results from this investigation show promise for adopting MPM-FLIM in the context of SLN diagnostics and encourage further translational studies on fresh tissue samples.

## 1 Introduction

Recent advancements in optical techniques such as multiphoton laser scanning microscopy (MPM),^1^^,^^2^ confocal reflectance microscopy,^3^ and optical coherence tomography^4^ have strengthened ongoing research targeting the development of optical tools that enable intravital tissue biopsy.^5^^,^^6^ MPM has been explored in the field of skin cancer research for the last few years and has demonstrated potential for use in facilitating diagnostics of primary malignant melanoma.^7^^,^^8^ Fluorescence lifetime imaging microscopy (FLIM) is a time-correlated fluorescence technique that generates images based on the fluorescence lifetime rather than intensity.^9^^–^^11^ FLIM has been applied to mapping the local environment of fluorophores such as ion concentration and pH sensing in various samples, since fluorescence lifetime depends on the molecular environment.^12^^,^^13^ Furthermore, FLIM can enable studies of metabolic activities in cellular environments by identifying the bound and unbound forms of nicotinamide adenine dinucleotide (NADH) and flavin adenine dinucleotide (FAD), two of the most common metabolic markers.^14^^,^^15^

According to the Global Burden of Disease Study in 2015,^16^ malignant melanoma processes a global incidence of 0.3 million, making it one of the most widely spreading skin cancers across the world. Sweden has the fourth highest number of melanoma incidents out of the 195 countries included in said study. Because of the high metastasizing potential of melanoma, early diagnostics are of utmost importance, and the five-year survival rate is more than 90% if melanoma is diagnosed in the early stage.^17^ The current clinical diagnostic practice for melanoma is to perform surgical excisions of the primary tumor. In the case of thick melanomas, it is followed by histopathological analysis of the sentinel lymph node (SLN), which is the first lymph node (LN) ascended by the metastatic cells.^18^^–^^20^ The histopathological analysis of the LN is time-consuming and requires arduous laboratory work. The accuracy of the SLN biopsy is not 100%; an erroneous diagnosis is inevitable.^18^^,^^21^^,^^22^ In addition, the removal of the LNs can lead to several complications such as wound infection, lymphedema, and hematoma.^23^^,^^24^ Therefore, the development of a technique that can assist surgeons in early metastasis staging is important. Until now, only a limited number of studies have investigated MPM for the purpose of melanoma metastasis diagnostics in LNs. In a recent study by our group, we observed that morphological features characteristic to melanoma metastasis can be discerned in LN tissues using MPM,^25^ but improved contrast would be required to facilitate diagnostics. Inspired by this work, we propose employing MPM combined with FLIM for investigation of primary melanoma lesions.^8^^,^^26^^,^^27^

The objective of this work was to investigate the potential for adopting MPM-FLIM to examine melanoma metastasis in SLN tissue. This was done by investigating differences between metastasized and nonmetastasized SLN tissue using MPM-FLIM *ex vivo*. The fluorescence lifetime from tissue inherent fluorophores was visualized and correlated with histopathological morphological features. To the best of the authors' knowledge this is the first study investigating melanoma metastasis in human SLN tissue *ex vivo* using MPM-FLIM.

## 2 Methods and Materials

### 2.1 Sentinel Lymph Node Tissues

In total, five LN tissue samples, three melanoma metastasis positive and two negative, were obtained from a biobank at the Department of Pathology, Sahlgrenska University Hospital, Gothenburg, Sweden, as a part of an ongoing study approved by the local ethics committee (University of Gothenburg, No 145-16). Detailed descriptions of samples and patient demography are provided (see Supplementary Material). Half of the intact tissue blocks were deparaffinized according to a customized protocol (see Supplementary Material) and stored in ethanol (70%, Sigma Aldrich) at room temperature to hydrate the samples. The other halves of the tissue blocks were processed for ordinary histopathological analysis. The deparaffinized tissues were mounted in customized imaging chambers, using ultrasound transmission gel (n=1.33, Parker laboratories) as the immersion media, before subjecting to MPM-FLIM.

### 2.2 Histopathological Analysis

Histopathological analysis of corresponding hematoxylin-eosin (H&E) stained glass slides was performed using a pathology slide scanner (Oncotopix scan, nanoZoomer, Visiopharm).

### 2.3 MPM-FLIM Imaging and Data Analysis

**Fig. 1:**
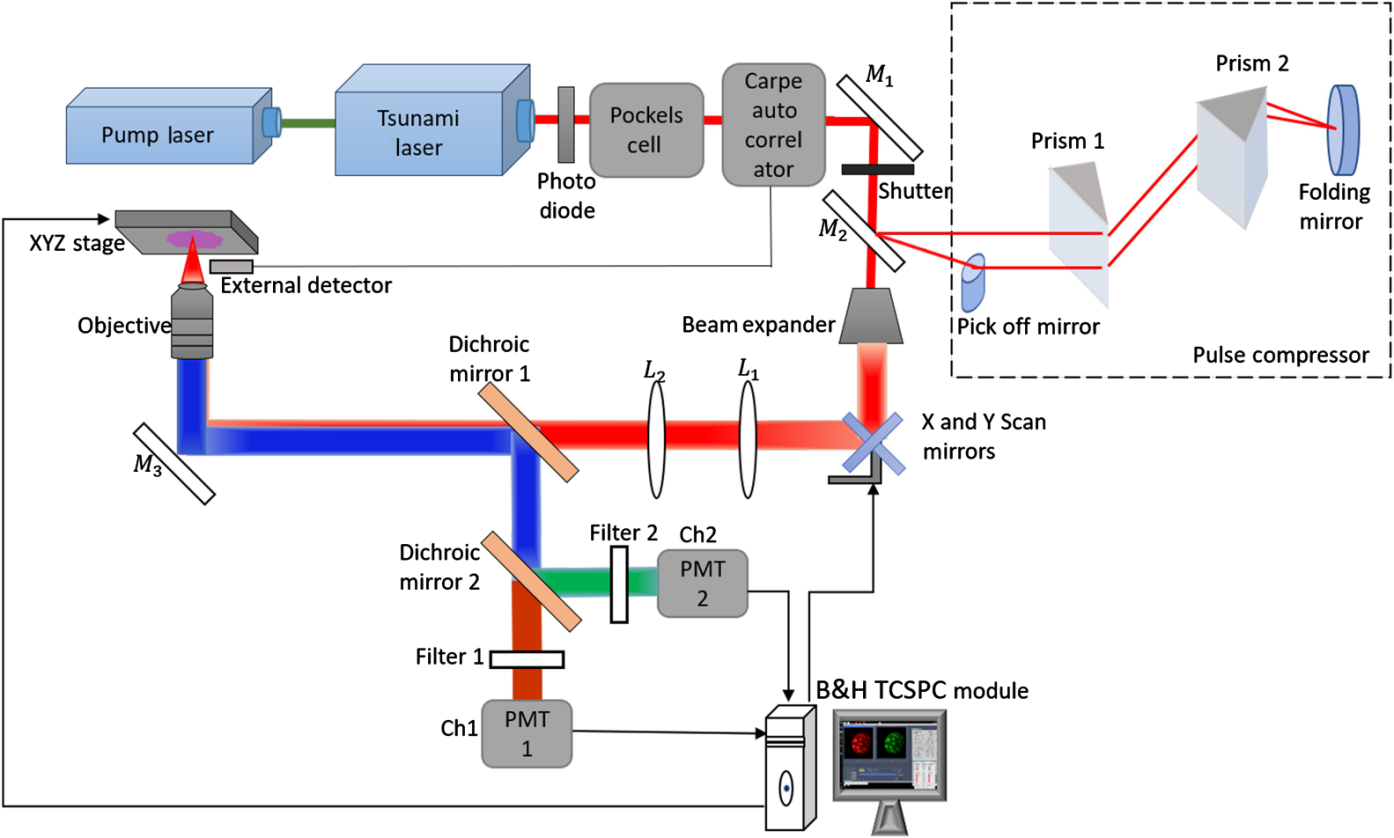
Schematic diagram of the experimental MPM set-up equipped with Carpe autocorrelator and femtocontrol pulse compressor for FLIM imaging.

The experimental MPM set-up is schematically illustrated in Fig. 1. Excitation was obtained by a fs-pulsed (∼80  fs) tunable (700 to 900 nm) Ti:Sapphire laser (Tsunami, Spectra physics) pumped by a frequency doubled Nd:YAG laser (Millenia, Spectra Physics, 532 nm). The output laser power was modulated by a Pockels cell (350-80LA, ConOptics). FLIM was recorded using an excitation wavelength of 780 nm, and average laser power <15  mW and 100 fs pulse duration at the sample plane were maintained by applying an autocorrelator (CARPE, APE) and pulse compressor (Femto control kit, APE).

As an objective lens, a water immersive C “Achroplan” NIR, 40×/0.8  W (Carl Zeiss) was utilized. The emission signal was detected by two GaAsP detectors (H7422P-40 MOD, Hamamatsu), interfaced to time correlated single photon counting modules SPC 150 (TCSPC, Becker&Hickl). Two spectral detection channels were set-up using a dichroic mirror (550 nm cut off, Semrock Inc.) combined with two filters 580/150  nm and 525/50  nm (Semrock, Brightline), enabling the two channels 1 (red, 550 to 655 nm) and 2 (green, 500 to 550 nm).

FLIM images were acquired at an area of 512×512  pixels, using pixel dwell time 1.8  μs and scanning a frame at a speed of 0.616 s. Total acquisition time for each image was around 60 s, including multiple frame scans, to increase the number of photons, which improved signal-to-noise ratio. FLIM analysis was performed using SPCImage 8 software (SPCM, Becker&Hickl).^28^ A single exponential decay model was applied to fit the fluorescence lifetime decays. FLIM data from different regions of interest were extracted from SPCImage, and the lifetime histograms were plotted in MATLAB (MathWorks Inc.).

## 3 Results

**Fig. 2:**
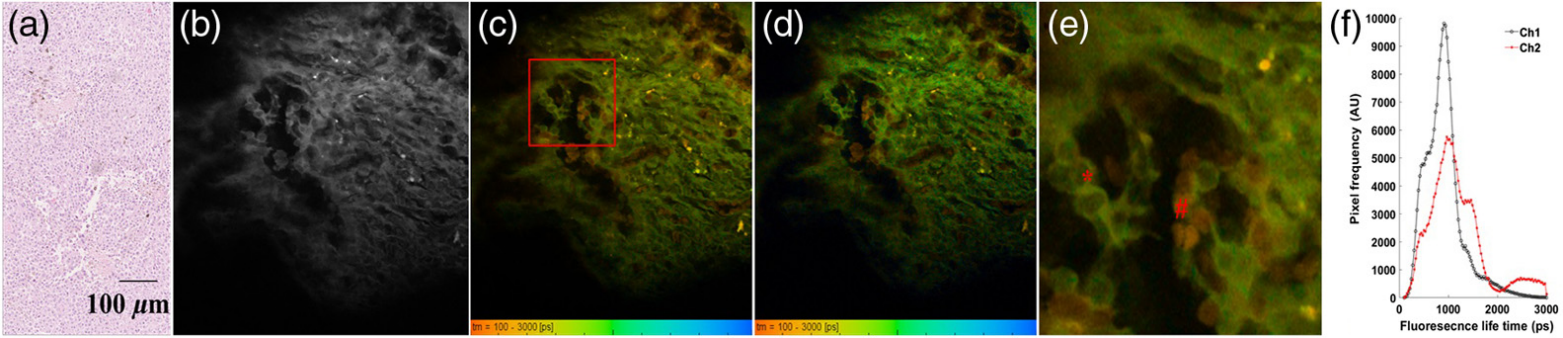
H&E stained histologic section (a) together with MPM intensity image (b); FLIM images obtained from channel 1, 580/150  nm (c), channel 2, 525/50  nm (d), of SLN (LN1) positive for melanoma metastasis and corresponding fluorescence lifetime distribution histogram (f) obtained from two spectral channels. The zoomed FLIM image (e) corresponding to the region of interest (highlighted red box) in (c). Atypical cells possibly undergoing mitosis (*) and randomly distributed cells (#) with short fluorescence lifetime (∼600  ps) are highlighted in (e). MPM and FLIM data acquired using 780 nm excitation. Field of view: (b)–(d) ∼250×250  μm and (e) 70×70  μm. False color scale lifetime data, 256-time channels, ranging from 100 to 3000 ps.

The results from this exploratory study report on the data acquired using MPM-FLIM *ex vivo* investigating five LN tissue samples, three melanoma metastasis positive and two negative. The data from all samples are presented in Figs. S1–S3 in the Supplementary Material. Figure 2 shows representative MPM intensity and FLIM images acquired from one of the melanoma positive tissues (LN1). Included in the figure is the corresponding image of an H&E stained tissue section from the same sample. As observed from this figure, the MPM intensity image [Fig. 2(b)] and FLIM images [Fig. 2(c), channel 1: 580/150  nm and Fig. 2(d), channel 2: 525/50  nm, respectively] display pleomorphism, i.e., varying cell sizes, similar to the H&E image [Fig. 2(a)]. Similar features were observed in all metastasis positive samples (Figs. S1 and S2 in the Supplementary Material). Overall, the values of the fluorescence lifetimes acquired from the samples are distributed around 100 to 3000 ps as shown in Fig. 2(f). The acquired lifetimes in both spectral channels most likely correspond to signals from NADH and FAD, but they are difficult to separate due to spectral cross-talk.^29^ The long fluorescence lifetime distribution >2000  ps in channel 2 [Fig. 2(f)] most likely corresponds to FAD, while the lifetimes in the range 300 to 2000 ps probably originate from NADH,^29^^–^^31^ which is also observed in channel 1. In the zoomed-in image [Fig. 2(e)], the cell structure becomes more evident; large atypical malignant cells (∼10  μm) display a fluorescent cytoplasm surrounding a prominent and less fluorescent nuclei. Highlighted in Fig. 2(e) is a possible mitosis (*), thus, supporting the presence of melanoma metastasis. In addition, cells exhibiting significantly shorter lifetime values ∼600 to 700 ps were also discerned, which can be seen in the orange color scale highlighted by (#). Based on their structure lacking visible nuclei, these cells most likely correspond to erythrocytes, which is further supported by findings discussed below.

**Fig. 3:**
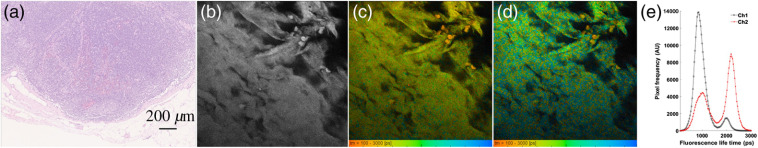
H&E stained histologic section (a) together with MPM intensity (b); FLIM images obtained from channel 1, 580/150 nm (c), channel 2, 525/50  nm (d) of a melanoma metastasis negative SLN (LN3) and fluorescence lifetime distribution histogram (e) obtained from both spectral channels. MPM and FLIM data acquired using 780 nm excitation. Field of view: (b)–(d) ∼350×350  μm. False color scale fluorescence lifetime data, 256-time channels, ranging from 100 to 3000 ps.

As a control, two melanoma negative SLN tissues (LN3 and LN5) were investigated (Fig. S3 in the Supplementary Material). The data presented in Fig. 3 demonstrate representative MPM-FLIM data together with a corresponding H&E stained tissue section of one of the melanoma negative SLN tissues (LN3). When comparing Figs. 2 and 3, it is evident that there is a clear difference in morphological structures between the samples. In the negative control sample, a more homogenous tissue matrix is visible, with hardly discernable small cells (∼7  μm) most likely representing lymphocytes, as represented in the corresponding H&E stained section. Interestingly, the distinct bimodal fluorescence lifetime distribution most likely corresponds to NADH and FAD in the two different spectral channels; the short lifetime corresponds to NADH free and the long lifetime probably conforms to both bound NADH and FAD.^29^^,^^31^ It is obvious that, when comparing the fluorescence lifetime data, the bimodal distribution is not present to the same extent in the malignant tissue (Fig. 2 and Fig. S2 in the Supplementary Material). Furthermore, the distribution between shorter and longer lifetime components is different in the two spectral channels. Thus the comparison of the lifetime distributions in the two different channels can potentially add spectral information to the morphological interpretation, thus facilitating diagnostics.

**Fig. 4:**
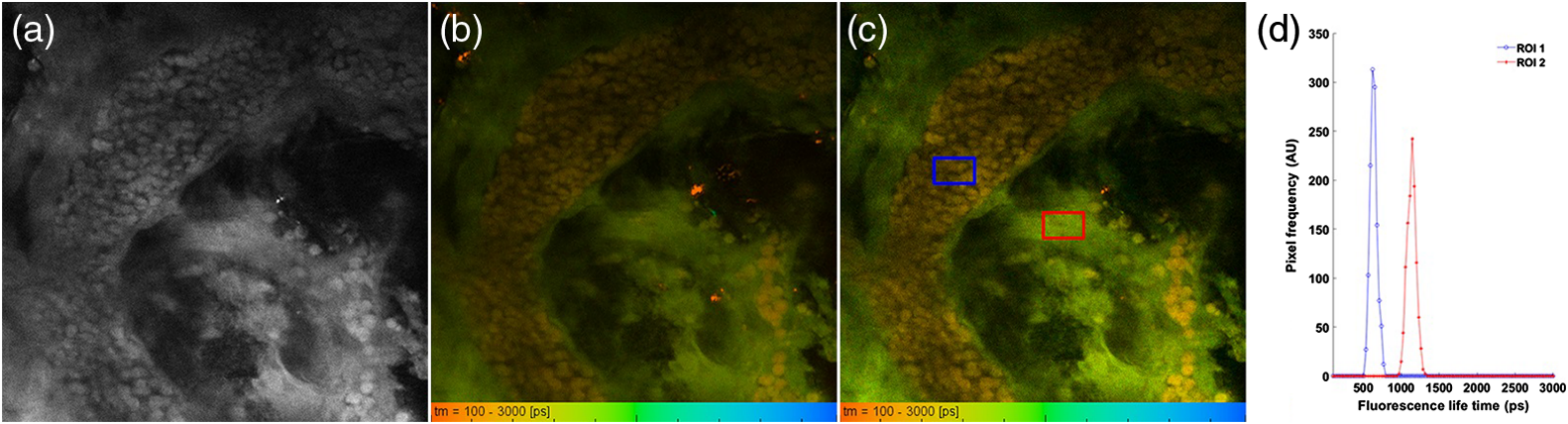
MPM intensity (a), FLIM images obtained from channel 1, 580/150  nm (b), and channel 2, 525/50  nm (c), of a positive SLN sample (LN2) and fluorescence lifetime distribution (d) obtained from different regions of interest as highlighted in (c). Field of view: 350×350  μm. False color scale fluorescence lifetime data, 256-time channels, ranging from 100 to 3000 ps. MPM and FLIM data are acquired at 780 nm.

The data in Fig. 4 represent MPM and FLIM images obtained from the metastasis positive LN2 sample, but from a different morphological region. As seen from the MPM intensity image [Fig. 4(a)], the morphological features are difficult to discern. However, turning to the FLIM images obtained from both spectral channels [Figs. 4(b) and 4(c)], completely different structures are revealed based on the lifetime. A structure of bright cells without clear nuclei and exhibiting short fluorescence lifetimes (∼600  ps) are clearly discerned in orange-red color. As this structure resembles a blood vessel, the interpretation is that the cells appearing in the orange-red color scale probably correspond to erythrocytes. This is more evident in Fig. 4(d), where the fluorescence lifetime distribution from the two different regions of interest is shown. The fluorescence lifetime distribution from the blood vessel (blue square) is centered around 500 to 800 ps, which is similar to the fast fluorescence decay of red blood cells as reported in the literature.^32^^,^^33^ By contrast, the lifetime distribution from the metastatic tissue region (red square) has 1000 to 1500 ps, which is analogous to the histogram from Fig. 2. Overall, these results indicate the potential of MPM-FLIM images to visualize the morphological features of positive and negative metastasized SLN tissue samples with better contrast features alongside the fluorescence lifetime information.

## 4 Discussion and Conclusion

Tools are required that can facilitate and complement histopathological analyses of LN tissues, particularly in connection to SLN diagnostics for cancer management, such as early metastatic staging of malignant melanoma. Recent work by our group has demonstrated the potential for employing MPM for investigating SLN tissue *ex vivo*,^25^ based on extracting LN tissue from a tissue biobank comprising tissues from melanoma patients having undergone SLN surgery at the local hospital. This biobank serves as a unique resource for exploratory studies aimed at the development of technologies targeting clinical translation. It was found that morphological features observed using MPM correlate with histology; however, further means to improve contrast are desirable. Thus, in this paper, we have extended these investigations to include FLIM analysis, using tissues from the same biobank. The intact tissue blocks were deparaffinized and subjected to MPM-FLIM *ex vivo* using an experimental MPM platform, complementary to the previous commercial MPM utilized in the previous work.^25^ Excitation was performed at 780 nm, and the emission was collected in red channel (ch1, 550 to 655 nm) and green channel (ch2, 500 to 550 nm), corresponding to the autofluorescence wavelength region expected for endogenous tissue chromophores, e.g., primarily NADH and FAD.^29^^–^^31^ In agreement with our previous study, morphological features signifying metastatic as well as normal lymphatic tissue were identified; however, contrast was enhanced and observed by highlighting morphologic features based on differences in fluorescence lifetime.

The bimodal lifetime distribution observed in the control (i.e., nonmetastatic) tissue samples was most likely interpreted in the context of free NADH and is reported to have a shorter lifetime (∼300  ps), and the bound NADH exhibit a longer lifetime (∼2000 to 2300 ps).^30^^,^^31^^,^^34^ In this study, the lifetime peaks were found around 1000 to 2300 ps (Fig. 3). Since the longer lifetime was more prominent in the green channel (channel 2), the signal most likely also comes from FAD, which is reported to have a lifetime ∼3000  ps.^15^ The shift in lifetimes for free NADH and FAD is most likely attributed to the fact that the investigated tissue has undergone substantial work-up by the preceding fixation and deparaffination process. It should be noted that the observed lifetime distribution of NADH and FAD is in agreement with earlier reports demonstrating increasing lifetimes (∼300 to 400 ps) in fixed tissues due to the different fixation protocols and mounting media.^35^^,^^36^

Also evident from the data was the identification of erythrocytes and blood vessels exhibiting short lifetimes ∼500 to 600 ps in the red channel and ∼600 to 800 ps in the green channel. It is reported that pure forms of haemoglobin and red blood cells in tissues have fast fluorescence decay (∼300  ps).^32^^,^^33^ However, contrary to these findings, a recent report on multiphoton excited haemoglobin in stored blood revealed a long-lifetime (∼1300  ps) component in addition to a short lifetime value (∼280  ps).^37^ The fluorescence lifetime values from our study are slightly higher than the values reported previously in the literature. This discrepancy could potentially be related to the metastasis microenvironment of the LN tissue, but it is most likely related to the preceding fixation and work-up of the tissue. Thus these discrepancies in lifetimes point to the necessity of confirming these results in fresh LN tissues to promote the MPM-FLIM technique for translation as an intravital tool for melanoma metastasis diagnostics.

From a morphological point of view, the FLIM-MPM adds an additional image contrast compared with the pure intensity data. For example, in this study, prominent features such as pleomorphism,^38^ atypical cells, and cellular mitosis were discerned. As the rate of mitosis is one of the factors used to diagnose and determine the stage of melanoma,^39^ this too is an important feature to look at. It is also known that the density of blood vessels is increased in metastatic tissue;^40^^–^^42^ thus the ability to clearly distinguish blood vessels and the presence of erythrocytes, as demonstrated (e.g., Fig. 4), is beneficial and adds further support for MPM-FLIM as a diagnostic tool for this type of tissue.

To conclude, this study demonstrates that MPM-FLIM-based on autofluorescence has the potential to visualize melanoma metastasis in human SLN tissue by being able to detect malignant atypical cells, healthy lymphocytes, blood vessels, and erythrocytes in the LN. Higher contrast is observed in the FLIM images in comparison with MPM intensity images, providing complementary morphological information. Further experiments on fresh LN tissues, are required to validate the approach as a diagnostic tool for early staging of malignant melanoma, potentially providing rapid histopathological analysis in conjunct with SLN surgery.
