# Supplementary material for: Report on fluorescence lifetime imaging using multiphoton laser scanning microscopy targeting sentinel lymph node diagnostics
Source: J Biomed Opt. 2020 Mar 14;25(7):071204. doi: 10.1117/1.JBO.25.7.071204 (PMC7070082; doi:10.1117/1.JBO.25.7.071204)
Supplement: Supplementary file 1 [file JBO_025_071204_SD001.docx]

**Supplementary materials**

**Report on fluorescence lifetime imaging using multiphoton laser scanning microscopy targeting sentinel lymph node diagnostics**

**Jeemol James^1, *^, Despoina Kantere^2^, Jonas Enger^3^, Jan Siarov^4^, Ann Marie Wennberg^2^ and Marica B. Ericson^1, *^**

^1^University of Gothenburg, Biomedical photonics group, Department of Chemistry and Molecular Biology, Gothenburg, Sweden

^2^University of Gothenburg, Institute of Clinical Sciences, Department of Dermatology and Venereology, Gothenburg, Sweden

^3^University of Gothenburg, Department of Physics, Gothenburg, Sweden

^4^University of Gothenburg, Department of Pathology, Gothenburg, Sweden

- **Supplementary methods**
- **Supplementary data**

- **Supplementary methods**

*Table S1. Demographic data and details of lymph node (LN) tissues included in the study.*

| **Patient No** | **Sample No** | **Year** | **Age at surgery** | **Gender** | **Location Primary MM** | **Histology Primary MM** | **Lymph node location** | **Confirmed SLN** | **Metastasis histologically confirmed in SLN** |
| --- | --- | --- | --- | --- | --- | --- | --- | --- | --- |
| **Patient 1** | LN1 | 2015 | 56 yrs | male | Back | MM nodular Breslow 2mm, Clark 3 | Left axil | SLN | yes |
| **Patient 2** | LN2 | 2012 | 31 yrs | male | Left thigh, above knee | MM nodular, 6 mm Breslow, Clark IV, with ulceration and mitoses | Left inguinal | SLN | yes |
| **Patient 3** | LN3 | 2013 | 56 yrs | female | Right arm | MM nodular, 3 mm Breslow, Clark IV, no ulceration | Right axill | SLN | no |
| **Patient 4** | LN4 | 2012 | 43 yrs | male | Back | SSM, Breslow 4 mm, Clark III, no ulceration, positive mitoses | Right axill | SNL | yes |
| **Patient 5** | LN5 | 2013 | 79 yrs | female | Left ankle | No specified MM, Breslow 4mm, Clark IV, no ulceration, positive mitoses | Left inguinal | SLN | no |

**Deparaffinization procedure**

The formalin-fixed and paraffin-embedded tissue blocks were cleared by four 15 min cycles in xylene (Histolab Products AB, ref: 02080, Sweden), followed by an additional four 15 min cycles in absolute ethanol (Histolab Products AB, Ref: 01399.25L, Sweden). The tissues were processed in a Vacuum Infiltration Processor (Tissue-Tek VIP6-E2, Sakura, Japan) using the so-called Clean Cycle program.

- **Supplementary data**

**Figure S1** represents MPM intensity and FLIM images obtained from LN2, i.e., MM positive SLN sample. As seen from the MPM intensity image (Fig S1.a) the morphological features are difficult to identify, while FLIM images obtained from both spectral channels (Fig S1.b and Fig S1.c) visualise different structures and features particularly atypical cells and erythrocytes. Structures of bright cells without nuclei exhibiting short fluorescence lifetimes (~ 600 ps, Fig S1.d) are clearly discerned in orange-red in FLIM images (highlighted by ‘*’). As discussed in main manuscript (Fig 2 and Fig 4), these cells most likely correspond to erythrocytes.


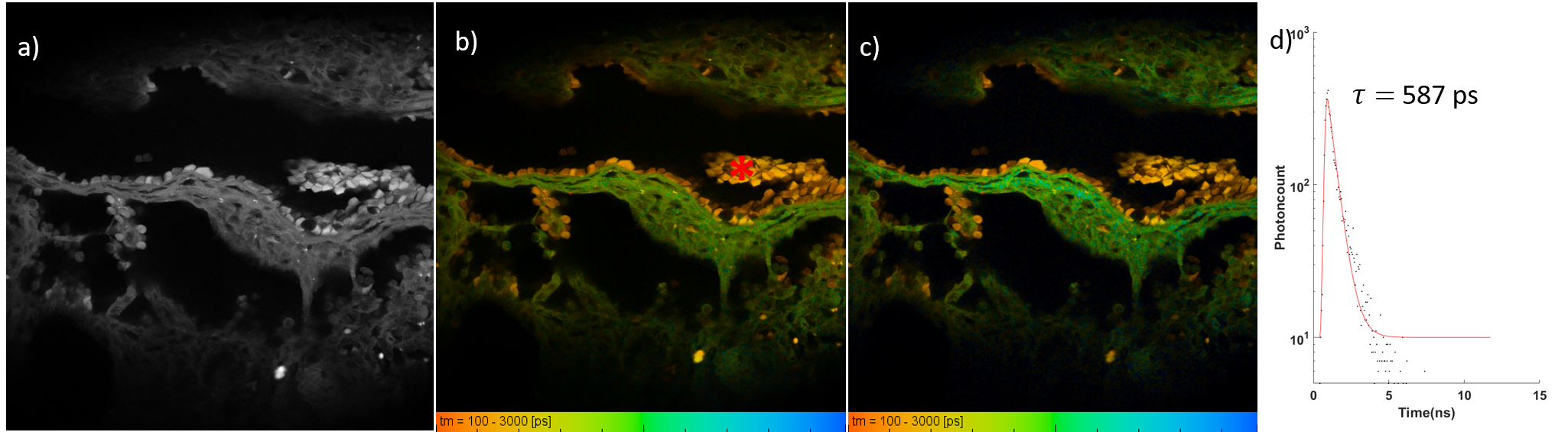


**Figure S1.** MPM intensity (a), and FLIM images obtained from channel 1, 580/150 nm (b) and channel 2, 525/50 nm (c) of a positive melanoma tissue (LN2) and corresponding fluorescence decay curve from highlighted (*) area is shown (d). Sequence of erythrocytes with short lifetime (~600 ps) value are visualised in both channels (b and c). Field of view: 350x350 µm. False color scale fluorescence life-time data, 256-time channels, ranging from 100 - 3000 ps. MPM and FLIM data were acquired at 780 nm.

**Figure S2** represents MPM intensity and FLIM images obtained from three positive melanoma SLN tissues extracted from two spectral channels. As observed in the FLIM images, large atypical cells are discerned in all the FLIM images as highlighted by red arrows. Also, bright erythrocytes and blood vessels, highlighted by green crosses, exhibiting short fluorescent lifetime values are visible in the FLIM images. This shows the potential of MPM-FLIM to identify atypical cells and other structures based on morphological features together with lifetime data.


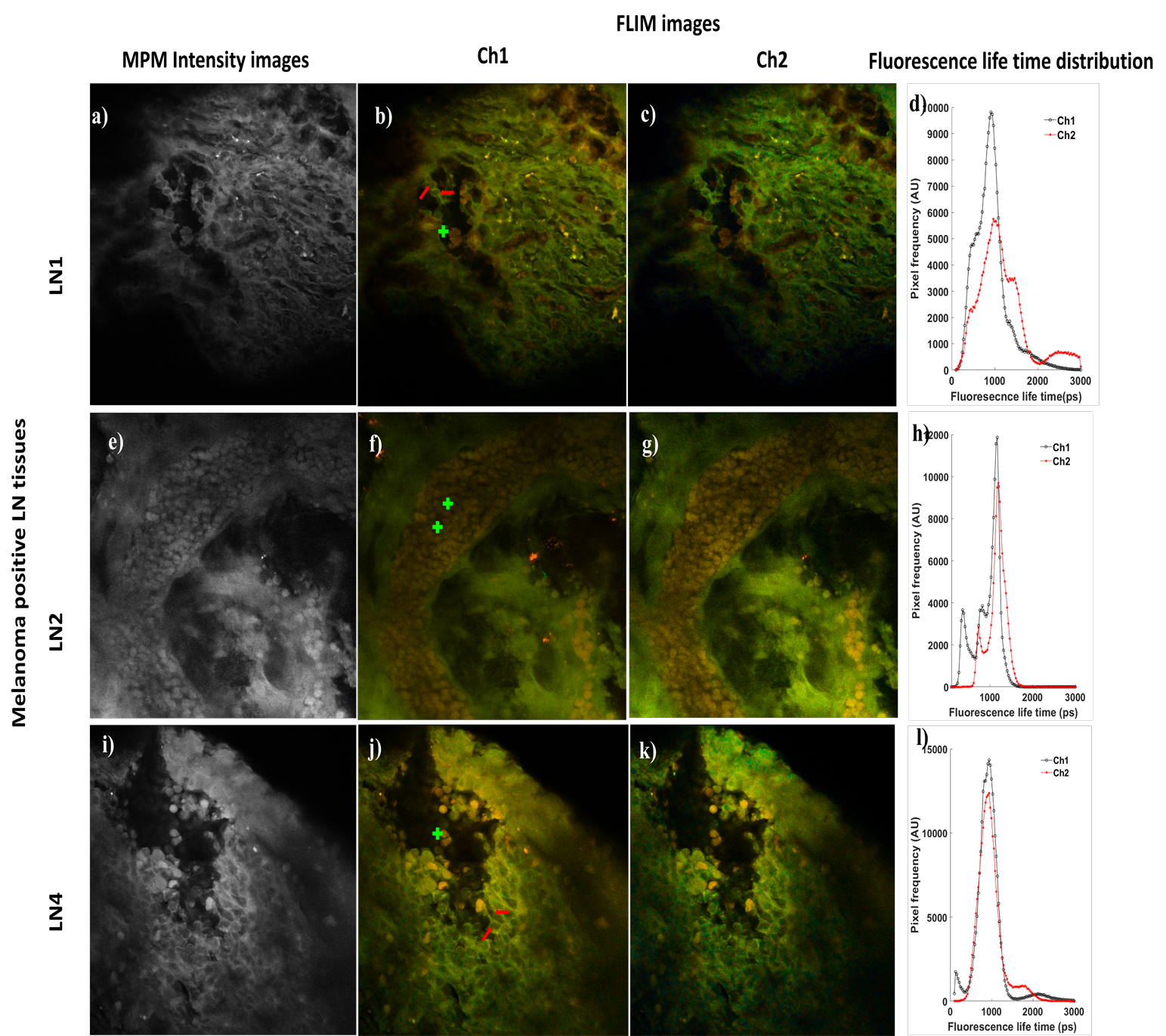


**Figure S2.** MPM intensity (a,e,i) and FLIM images (b,c,f,g,j,k) of three positive melanoma (LN1, LN2 and LN4) tissues obtained from two spectral channels (Ch1- 580/150 nm and Ch2-525/50 nm) along with corresponding fluorescence life time distribution (d,h,l). Atypical cells (arrows, red color), erythrocytes and blood vessels (cross, green color) are highlighted in FLIM images (b,f,j). Field of view: (a,b,c) ~250x250 µm and (e,f,g,i,j,k) ~350x350 µm. False color scale fluorescence life-time data, 256-time channels, ranging from 100 - 3000 ps. MPM and FLIM data were acquired at 780 nm.

**Figure S3** represents MPM intensity and FLIM images obtained from two metastasis negative tissues (LN3 and LN5) extracted from two spectral channels. As can be seen from the figure, the metastasis negative SLN tissues have a homogenous morphological structure as compared to the metastasised tissues. The fluorescence lifetime histogram follows a bimodal distribution most likely corresponding to NADH and FAD is shown (d,h,l).


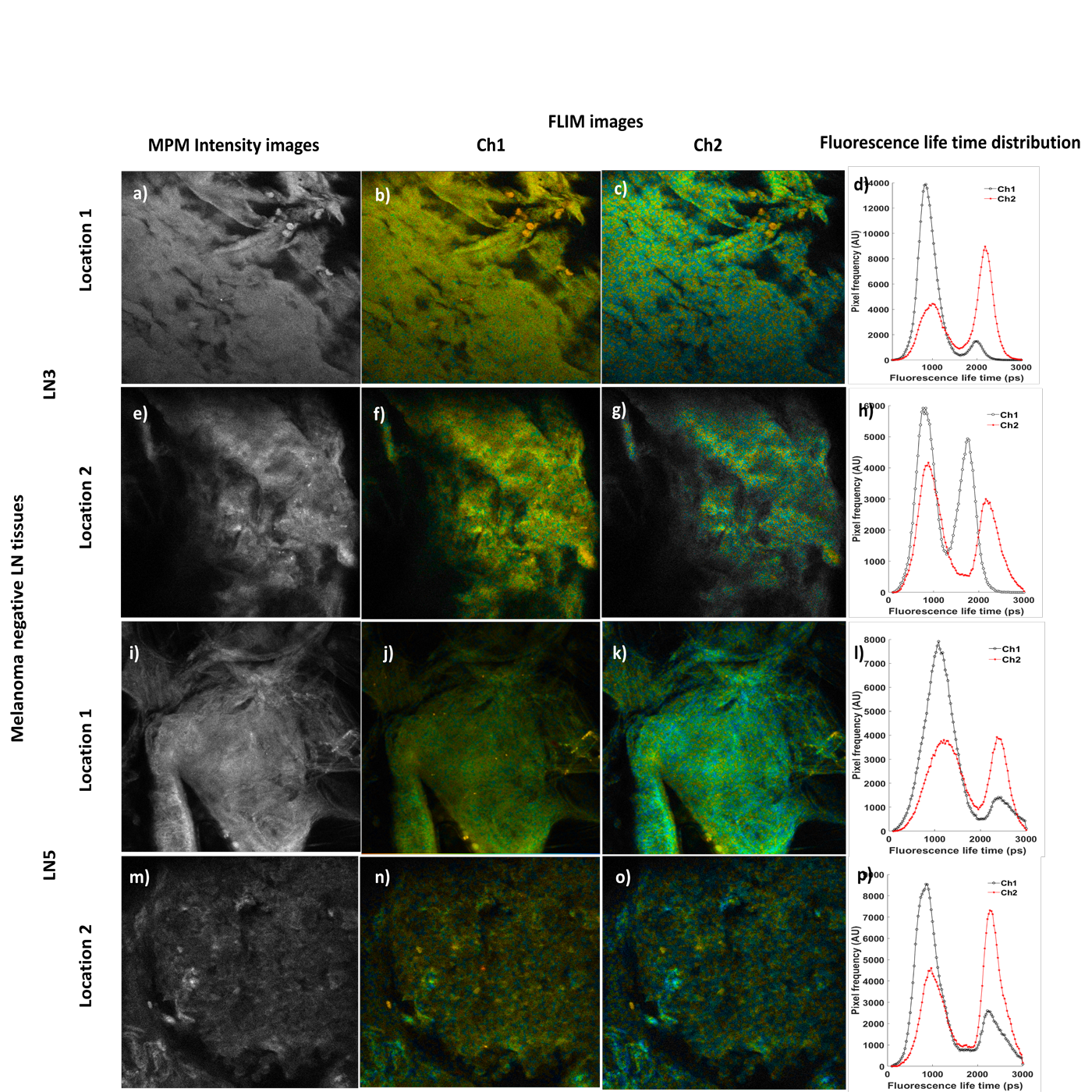


**Figure S3.** MPM intensity (a,e,i) and FLIM images (b,c,f,g,j,k) of two melanoma negative (LN3 and LN5) tissues obtained from two spectral channels (Ch1- 580/150 nm and Ch2-525/50 nm) along with corresponding fluorescence life time distribution (d,h,l). ). Field of view: ~350x350 µm. False color scale fluorescence life-time data, 256-time channels, ranging from 100 - 3000 ps. MPM and FLIM data were acquired at 780 nm.
